# RETRACTION: Negative Pressure Wound Therapy Compared with Conventional Wound Dressings for Closed Incisions in Orthopaedic Trauma Surgery: A Meta‐Analysis

**DOI:** 10.1111/iwj.70419

**Published:** 2025-03-26

## Abstract

**RETRACTION:** XieW. , DaiL. , QiY. , and JiangX. , “Negative Pressure Wound Therapy Compared with Conventional Wound Dressings for Closed Incisions in Orthopaedic Trauma Surgery: A Meta‐Analysis,” International Wound Journal 19, no. 6 (2022): 1319–1328, 10.1111/iwj.13726.34854236 PMC9493229

The above article, published online on 02 December 2021, in Wiley Online Library (http://onlinelibrary.wiley.com/), has been retracted by agreement between the journal Editor in Chief, Professor Keith Harding; and John Wiley & Sons Ltd. Following an investigation by the publisher, all parties have concluded that this article was accepted solely on the basis of a compromised peer review process. The editors have therefore decided to retract the article. The authors did not respond to our notice regarding the retraction.

**RETRACTION:** W. Xie , L. Dai , Y. Qi , and X. Jiang , “Negative Pressure Wound Therapy Compared with Conventional Wound Dressings for Closed Incisions in Orthopaedic Trauma Surgery: A Meta‐Analysis,” International Wound Journal 19, no. 6 (2022): 1319–1328, 10.1111/iwj.13726.34854236 PMC9493229

The above article, published online on 02 December 2021, in Wiley Online Library (http://onlinelibrary.wiley.com/), has been retracted by agreement between the journal Editor in Chief, Professor Keith Harding; and John Wiley & Sons Ltd. Following an investigation by the publisher, all parties have concluded that this article was accepted solely on the basis of a compromised peer review process. The editors have therefore decided to retract the article. The authors did not respond to our notice regarding the retraction.

